# Functional Gastrointestinal Disorders with Psychiatric Symptoms: Involvement of the Microbiome–Gut–Brain Axis in the Pathophysiology and Case Management

**DOI:** 10.3390/microorganisms10112199

**Published:** 2022-11-07

**Authors:** Cristina Gabriela Șchiopu, Cristinel Ștefănescu, Alexandra Boloș, Smaranda Diaconescu, Georgiana-Emmanuela Gilca-Blanariu, Gabriela Ștefănescu

**Affiliations:** 1 Department of Psychiatry, University of Medicine and Pharmacy “Grigore T. Popa”, 700115 Iași, Romania; 2 Medical-Surgical Department, Faculty of Medicine, University “Titu Maiorescu”, 040441 Bucuresti, Romania; 3 Department of Gastroentereology, University of Medicine and Pharmacy “Grigore T. Popa”, 700115 Iași, Romania

**Keywords:** functional, microbiome, gut–brain axis, probiotics, psychiatry, gastroenterology

## Abstract

Functional Gastrointestinal Disorders have been an important cause of poor life quality in affected populations. The unclear etiology and pathophysiological mechanism alter the clinical evolution of the patient. Although a strong connection with psychological stress has been observed, it was not until recently that the gut–brain axis involvement has been revealed. Furthermore, the current literature not only promotes the gut–brain axis modulation as a therapeutical target for functional digestive disorders but also states that the gut microbiome has a main role in this bi-directional mechanism. Psychiatric symptoms are currently recognized as an equally important aspect of the clinical manifestation and modulation of both the digestive and central nervous systems and could be the best approach in restoring the balance. As such, this article proposes a detailed description of the physiology of the microbiome–gut–brain axis, the pathophysiology of the functional gastrointestinal disorders with psychiatric symptoms and current perspectives for therapeutical management, as revealed by the latest studies in the scientific literature.

## 1. Introduction

Human microbiome has not been at the center of scientific research until recent years, when the scientific approach to the gut–brain axis and its medical involvement in multiple pathologies has revealed the decisive role of the intestinal flora.

The most important implications for the microbiome, and the gut–brain connection, reside within the Functional Gastrointestinal Disorders (FGID) which have been recently linked to psychiatric symptoms. Interestingly, it seems that for the majority of patients, no matter the primary complain, whether it is psychiatric or gastrointestinal, the other end of the axis will clinically manifest at some point. This is the main reason why these cases often had poor therapeutical response until recently [1].

Although the scientific literature is evolving and clinical management has already improved, the research has several answers to reveal. For the time being, we propose a literature review that will cover the theoretical knowledge over the microbiome–gut–brain axis and its involvement in functional gastrointestinal pathology with psychiatric symptoms, for both the pediatric and adult population, starting from the physiology and pathophysiology and continuing with clinical and therapeutical aspects, in light of the current state-of-the-art.

## 2. Materials and Methods

The present paper proposes a literature review that focuses on the management of functional gastrointestinal disorders associated with psychiatric symptoms, by modulating the gut–brain axis, in both the adult and pediatric population. Additionally, the role of the microbiome within the bidirectional function of the gut–brain axis is of major importance. As such, the therapeutical management of dysbiosis, association with other pharmacological approaches and their impact over the digestive and psychiatric symptoms will be described.

Specific literature was selected from three databases: Elsevier, PubMed and ReasearchGate. Keywords included: gut–brain axis, functional gastrointestinal disorders, psychiatric symptoms, microbiome, intestinal metabolome, prebiotics, probiotics, psychobiotics, combining the terms in multiple variants. Initially, the search was refined by selecting articles written in the last 3 years but that led to a small amount of results and evaluating secondary documents led to finding articles dating back to 2005 that were selected, in order for the subject of the article to be rightfully developed and argued. The research included open access articles for the clinical and therapeutical features, featuring both animal and human studies. Extended literature research, including articles and books, was carried out to provide the current state-of-the-art regarding the physiology and physiopathology of the gut–brain axis. Review articles were added to the research for a detailed description of the theoretical baseline. ROMA IV criteria were used in order to approach FGIDs as the primary pathology with secondary psychiatric symptoms but also, primary psychiatric disorders with digestive functional symptoms were followed throughout the literature.

## 3. Review of Gut–Brain Axis and the Microbiome Physiology

A perfect and balanced coordination between the central nervous system and the enteric environment requires a complex and delicate calibration involving sympathetic and parasympathetic relays, hormonal and neurotransmitter activation and the autonomous nervous function. Through these pathways, there is a bidirectional regulation and influence over cognitive, emotion, behavior on one side and the functionality of the gastrointestinal system, with all roles involved: digestion, absorption and enteric immune system [2,3]. The proportion and influence level that the two systems exert on each other are still subjects of debate but it is clear that they influence each other by efferent and afferent pathways and the physiologic balance is affected heavily by outer factors, both from outside and inside the body. Beyond the direct relationship between central nervous system and gastrointestinal system, there are outer elements that influence the axis in negative or positive proportions, such as nutrients, pharmacological treatments, social and environmental factors, stress, behavior, mood and genetics [4].

### 3.1. The Microbiome

Most of the present literature supports the theory that describes the gastrointestinal microbiome and its specific strains as a main modulator of the gut–brain axis, acting decisively in functional gastrointestinal pathophysiology with manifesting psychiatric features. The hypothesis begins from simple clinical observations of antibiotic-treated patients with hepatic encephalopathy and afterwards, studies on germ-free animals have further demonstrated the importance of the microbiome in the development and physiology of the central nervous system. Nowadays, there is no doubt about the major implications of the microbiome in the homeostasis of the gut–brain axis and its involvement in many more medical fields, especially psychiatry and neurology. Furthermore, it seems that every species of bacteria has a specific function such as maintaining the intestinal barrier integrity [5], modulating afferent sensory nerves’ calcium-dependent potassium channels [6], promoting local neurotransmitter precursors for GABA, serotonin, acetylcholine [7], stimulating the sympathetic nervous system through bacterial metabolites such as butyric acid, propionic acid or acetic acid [8] and lastly, mediating mucosal immune activity through the enteral nervous system, P substance and proteases [9]. Additionally, the mechanism through which the microbiome influences the gut–brain axis mechanism is related to the vague nerve [10]. The present article, however, focuses on the microbiome and the gut–brain axis functions related to functional digestive disorders and coexisting psychiatric symptoms.

As such, modulating the composition of the gut microbiome and identifying its specific strain disbalance may be a future target in the management of functional gastrointestinal diseases and neuro–psychiatric-associated pathology. [11].

Given the current knowledge, the best definition of the interactions between the GI tract and the brain is the microbiome–gut–brain axis, as a recognition for the important role of the microbiome. Although the interaction per se is a synergic mechanism that modulates multiple physiological variables, it is important to understand the influence of every direction, as most new hypotheses state that every pathway of the axis is a key for unlocking therapeutic targets in different diseases [12].

### 3.2. Brain to Gut Connection and Gut to Brain Connection

The interaction between the central nervous system and the intestinal environment is based on neuronal, hormonal, immune and metabolic reactions. On one hand, the central nervous system is capable of influencing the local enteral nervous system, activating the hypothalamic pituitary adrenal axis and signaling through the vague nerve which will alter enteral environment through mucus production, permeability of the intestinal wall and immune reactivity that will further alter the microbiome strain population, causing more enteral disbalance that will redirect pathologic signals back to the brain. As such, the specific separate role of each efferent and afferent pathway and their synergy function with the microbiome is vital for understanding its pathophysiology [13].

The individuality of the two pathways and the proportions of each function inside the axis were described in a study in 2016 led by Keightley, that observed patients with functional digestive disorders and psychiatric symptoms. The study separated the patients into two groups, by baseline complaints and symptoms and the clinical aspects that appeared at 1 year follow-up. The group with digestive primary complaints had significantly more anxiety and depressive symptoms at 1 year follow-up and the majority of the patients with anxious and depressive baseline complaints had developed irritable bowel syndrome or other functional digestive symptoms at 1 year follow-up. Additionally, the most important changes were in the digestive baseline symptoms’ group, which suggest higher afferent influence than on the efferent pathway. These findings suggest that the gut–brain axis can be analyzed individually, by starting from each end, but also, that the gut and microbiome could exert more influence on the brain than initially thought [14].

### 3.3. Functional Mechanisms and Pathways between Microbiome, Gut and Brain

The three centers of the axis work individually as well as in a synergic cooperation keeping the sensitive balance with the help of nervous, immune and endocrine systems. Each physiologic pathway is a possible therapeutic target and should be assessed as a specific as well as integrated mandatory part of the whole mechanism [15].

#### 3.3.1. The Autonomous Nervous System

The autonomous nervous system maintains the homeostasis of the GI tract by managing endocrine, motor and behavioral signals by linking both brain and digestive systems. The autonomous nervous system controls the information received from the central nervous system and neuro–endocrine axis and assures intestinal responses in the form of permeability, motility, mucosal state and enteral immune responses, this being the efferent way. On the afferent path, distress, dysbiosis, and pain local signals are modulated and transmitted to the central nervous system via sympathetic and parasympathetic system, triggering responses in the connected cerebral areas [16]. Furthermore, it has been demonstrated that the microbiome is capable of interaction with the autonomous nervous system through microbial metabolites that act similar to excitatory or inhibitory triggers on the sympathetic and parasympathetic network. Tryptophan, catecholamines and serotonin have direct influence over the afferent pathways, inducing mood or cognitive changes.

The main communication path is apparently the vagus nerve, acting similar to a two-way highway, that is supposed to transport signals directly from microbiome and local alteration stimuli but also directing responses from the brain. A study in 2005 was relevant in this direction as it demonstrated the direct activation of vagal ganglia and relay nucleus in the medulla oblongata after Campylobacter gut inoculation in mice, followed by anxiety-like behavior. The study was relevant for future therapeutic modulation of the gut–brain axis through its fastest route that is the vagus nerve [17]. Vagal afferents transmit information from the gut with decreasing fiber density from the duodenum to the transverse colon with terminal connectivity in the lamina, soft muscle, mucosa and in some of the neuroendocrine cells. The gut level synapses are capable of detecting all alterations in the intestinal environment, each afferent having specific roles and, translating specific responses from the nervous system [18]. Modulating inflammatory responses and mood/affective responses to local gut dysfunction are also demonstrated to be managed by the vagus. The most relevant observations in this direction were the studies on vagotomized animals and humans. Vagotomy, as part of peptic ulcer treatment was reported to raise the incidence of psychiatric disorders in the studied population versus control In addition, probiotics have been studied recently as adjuvant therapy in anxiety and depression with promising results but it has been observed that positive results due to administering *Lactobacillus rhamnosus* are not the same in vagotomized animals compared to controls [19].

#### 3.3.2. The Enteric Nervous System

One of the most important relays in the gut–brain connection consists of the enteral nervous system. It is capable of acting independently or as a part of the autonomous nervous system. As part of the brain–gut axis, it serves as a transmitter of information to the brain via vague nerve afferents and given the majority of afferent fibers, it is presumed that this pathway of the axis acts more like a transmitter. Moreover, it is known that the enteric nervous system can function independently if the vague connection to the brain is severed, continuing to manage local bowel mechanisms and homeostasis [20]. In a physiologic environment, the local bowel disruptions will signal the brain and will turn on the vago–vagal reflexes which will regulate motility, mucosal functions and even microbiome balance. The enteric nervous system also transmits satiety or nausea sensations to the central nervous system which, apparently, bypasses consciousness but still, alters mood behavior and cognition. Given the above, the modulation of the gut–brain axis should approach the enteric nervous system as much as an independent mechanism and as an important relay of the gut–brain axis. The enteric nervous system is currently under study as it may be involved in neuro-degenerative disorders but also in spectrum disorders as autism has the most GI comorbidities amongst primary psychiatric diseases [21].

#### 3.3.3. The Enteric Immune System

The interface between microbiome and intestinal tissue consists of a dense mucosal layer that acts as a protectant but also as a center for coordination between internal environment and the intestinal lumen. At this level, there is a mandatory immune mechanism to manage that interface and maintain a synergic collaboration between intra-luminal and extra-luminal systems. As such, the enteric immune system must recognize self-antigens and eliminate potential harmful microorganisms. Moreover, the intestinal epithelium contains various cells that can trigger immune responses and release pro-inflammatory substances. [22] The immune modulation of the gut–brain axis resides in the microglial activity. First of all, connections between microglia and the microbiome are scarcely studied but there seems to be a strong belief that the microbiome is modulating the microglial development and activity [23]. Furthermore, the strong connection between microglia and microbiome was demonstrated in an animal model study, in which germ-free mice were exposed to a neurotropic virus. The germ-free mice were unable to stimulate anti-viral immunity and the lack of microglial activity led to demyelination. The physio–pathologic process was still reversible as the mice’s intestinal environment was repopulated by healthy microbiome [24].

Microbiome modulated microglial activity in the gut–brain axis consists of triggering immune responses, on the one hand, through a general immune response and especially, monocytes and further through TNF-α, interleukins and immunoglobulins and, on the other hand, through specific immune responses through lymphoid cells [25,26]. Studies highlight the role of microbiome in managing neuroimmune responses. As such, it seems that CD4 and CD8 lymphocytes were reprogrammed into T-cells and used as immunoregulators following L. reuteri treatment that generated tryptophan derivates and activated specific receptors in CD4. Of course, the research is in animal model stages but results are relevant and bring new perspectives upon neuro-immunity and microbiome importance within the gut–brain axis [27]. Even more so, lymphocyte deficiency resulted in cognitive disruptions and anxiety symptoms that were treated with lactobacillus species probiotics. This is yet another statement in favor of the synergy between neuro-immunity mechanisms inside the gut–brain axis and the microbiome [28].

#### 3.3.4. The Neuroendocrine System

The neuroendocrine enteric system seems to be connected to the enteric nervous system and to the microbiome, being currently under research for its possible therapeutic implications in metabolic diseases, especially in the type II diabetes. Neuroendocrinal representatives are the entero-chromaffin cells and the enteroendocrine L cells. Both types of cells are dispersed throughout the distal small intestine and colon, as well as the majority of the flora resides but also, they make contact with most of luminal constituents and as well, with enteric nervous and immune systems. The interactions with the microbiome are still under study and not yet clear but it is certain that microbial metabolites can trigger neuroendocrine responses through its specific cells [29].

Enterochromaffin cells were demonstrated in animal studies to interact and respond to microbial metabolites in the colonic lumen by expressing and activating 5-hydroxytryptamine (5-HT), from tryptophan and signal afferent vagal pathways, modulating peristatic function, pain or inflammation [30]. Moreover, another study revealed that increasing the population of Clostridium could elevate the 5-HT expression and accelerate intestinal transit. The ideas of this study are currently investigated for possible Crohn’s disease future research [31]. The enterochromaffin cells’ activity is not likely to impact the brain directly but it could interact with the gut–brain axis via vagal fibers as 5-HT does not cross the blood–brain barrier. The indirect impact of 5-HT has been demonstrated in chemotherapy as nausea and vomiting are produced by massive discharge of 5-HT and altered signaling in the gut [32]. Irritable bowel syndrome seems to undergo the same altered 5-HT expression as the new hypotheses state [33].

Enteroendocrine L cells are responsible for the expression of YY peptide (PYY) and glucagon-like peptide (GLP-1). The receptors for these are widely expressed throughout the gut and central nervous system, involving even the hypothalamic axis. The role of this system is transmitting information about food intake and satiety to the brain. Given the potent connection to the central nervous system, the peptides secreted by enteroendocrine L cells are known to have major implications in eating disorders, especially anorexia [34]. If in the proximal area of the gut, the enterochromaffin cells respond to nutrients and activate peptide release, in the distal it seems there are L cells that are almost entirely engaged in bacterial metabolite interactions [35]. The role of the neuroendocrine peptides in eating disorders and metabolic diseases and its interactions with the microbiome is gaining interest in the research field. As studies have stated, prebiotic (polysaccharides) and probiotic (Lactobacillus) supplementation results in increasing levels of GLP-1 and PPY with further reduction of food intake and insulin resistance [36,37].

### 3.4. The Hypothalamic–Pituitary–Adrenal Axis (HPA Axis)

The HPA axis is a key player within the gut–brain axis as it reacts to both brain and gut signals and assures a bidirectional response to stress. Studies have revealed an important role of the HPA axis in the gut environment of pediatric patients, a well as infants and children [38]. In animal models and human studies, prebiotic therapy altered the HPA-axis response, probably by indirect manipulation of gut microbiome [39].

### 3.5. Spinal Cord within the Gut–Brain Axis

The spinal cord acts like a signal carrier for pain and distress to the brain, via spinothalamic, spinomesencephalic and spinoreticular tracts. The routes conduct location and intensity and nociceptive signals that will be processed in emotional and behavioral areas of the brain and furthermore, respond with excitatory or inhibitory signals [40]. Following brain injury models, studies have demonstrated a bidirectional coordination between spinal cord and gut microbiome with mutual altering responses at distress stimulation; more specifically, spinal cord lesions promote microbiome imbalances that will maintain a negative feedback on the spinal cord [41].

### 3.6. The Neurotransmitters

Neurotransmitters have been studied for a long time as they are supposed to be the main linkage between functional digestive symptoms and neuropsychiatric disorders, affecting the digestive mechanism and mood, behavior and cognition at the other end.

Within the gut–brain axis, there is a strong crosstalk with the microbiome as gut bacteria are capable of producing β-glucuronidase that activates dopamine and epinephrine. In addition, catecholamines can be directly produced by some species of bacteria such as Bacillus and by doing so, enhance the neuroendocrine communication between gut and brain [42].

GABA is an important inhibitory neurotransmitter that acts within the central nervous system. GABA production in the intestinal area is linked to Lactobacillus which appears to be capable of GABA synthesis. This mechanism is currently under study as neurotransmitter productive probiotics could be used in treating neuro-gastrointestinal disorders [43].

Serotonin has been highly studied as a pathophysiologic background of the gut–brain axis mediated psychiatric symptoms. The production of serotonin in the central nervous system is linked to sleep, mood and appetite and the serotonin produced by entero-chromaffin cells in the intestinal tract has roles in motility and inflammation. However, given the gut–brain axis crosstalk, and the functions this neurotransmitter meets, there is no doubt the serotonin levels via the gut–brain axis are important for modulating mood disorders associated especially with functional digestive symptoms. Animal model studies have demonstrated that serotonin levels are much higher in rich diversified flora mice than germ-free mice [44]. Even more interestingly, germ-free mice have also low hippocampus serotonin levels contrasting with rich tryptophan circulating levels, which is a serotonin precursor. Metabolism of tryptophan is managed by gut flora and being a precursor of serotonin but also of other neurotransmitters involved in neuroendocrine and immune responses. As such, serotonin and probiotic therapy in neuro-gastroenterology are clear options but usage of serotonin directly or its precursor is what studies are debating [45].

Histamine, beyond its immune role, can be produced by enterochromaffin cells and histamine activity within the intestinal tract is linked to intestinal inflammation and luminal integrity. In addition, species of the microbiome are able to also produce histamine. In vitro studies have revealed that *Lactobacillus reuteri* has histamine producing capacity which decreases the levels of TNF-α, followed by reduction of intestinal inflammation via H2 receptors which raise new hypotheses targeting histamine in inflammatory bowel diseases [46,47].

### 3.7. Amino Acids and Microbiome

Beyond the critical role of amino acids within the human body, even more important are the essential amino acids that cannot be produced by the body but need to be synthetized from dietary intake These are the branched chain amino acids that are valine, leucine and isoleucine. The microbiome is more specialized in producing branched chain amino acids more than other amino acids. Animal studies have revealed that a restrictive diet and administration of branched chain amino acids increases the bacterial growth of the Bacteroides species more than other species, followed by decreased intestinal inflammatory processes [48,49].

### 3.8. Microbial Metabolism of Short Chain Fatty Acids

The gut microbiome’s basic roles is the metabolism of complex nutrients and the transformation of complex chemical substances. Nutrients are reduced to simple sugars and fermented down to fatty acids with short chains, with abundance in anaerobic population areas. Short chain fatty acids are acetic, butyric, propionic or lactic acid. Short chain fatty acids also have roles in increasing absorption of vitamins and minerals such as calcium, iron and magnesium and help with glucose and protein liver metabolism. They maintain the integrity of structure and function of the intestinal tract assuring efficient digestive function. Furthermore, they are metabolized as an energy source via the Krebs cycle in the cell environment [50]. Apart from the metabolic and immune roles of short chain fatty acids, influence of the gut–brain axis involves the neuroendocrine axis via acetate modulation of the hypothalamic function, as acetate as well as other acids are capable of reaching the brain by the blood–brain barrier and moderate the activity of neuropeptides responsible for appetite control [51,52]. More relevant and interesting studies conducted on animal models demonstrate that short chain fatty acids could directly influence the brain as they could affect brain areas such as the hippo-campus and striatum, influencing cognitive functions, memory learning and reward-associated behavior (developmental or addictive-like). Moreover, diet supplementation of short chain fatty acids resulted in reduced anhedonia, anxiety and depressive disorders in mice, interestingly accompanied by decreased corticoid receptors in both hypothalamus and colon [53]. Implications of short chain fatty acids in neuropsychiatric disorders are already under heavy research as primary findings offer positive perspectives. High level intra-cerebral, specifically intra-ventricular short chain fatty acids, are supposed to be involved in the pathophysiology of Parkinson’s Disease or even in autistic spectrum disorders and epilepsy [54].

**Figure 1:**
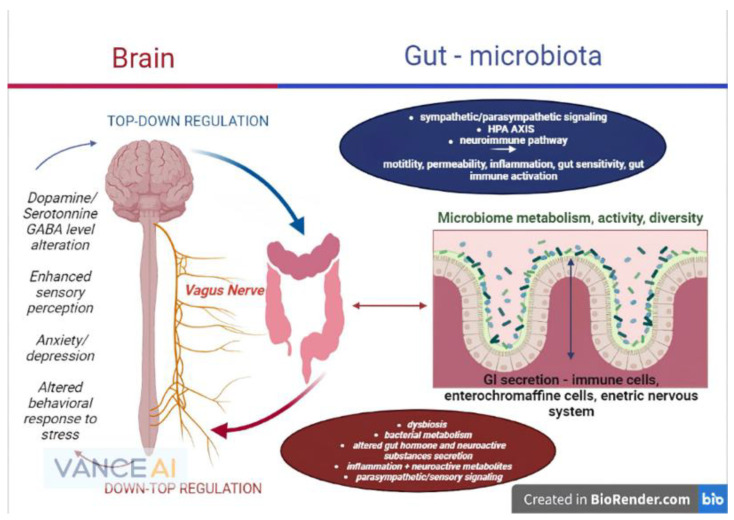
The microbiome–gut–brain axis and its physiopathological interconnections (remake after https://www.frontiersin.org/files/Articles/813204/fmed-09-813204-HTML-r1/image_m/fmed-09-813204-g001.jpg (accessed on 20 September 2022)).

In light of the statements above, future research on all branches of the gut–brain axis should focus primarily on the microbiome and its critical role as a homeostasis mediator between the brain and gastrointestinal environment, as the majority of the mechanisms and reactions of the gut–brain axis are secondary to the gut bacteria’s functions (Figure 1).

## 4. Development and Importance of Microbiome–Gut–Brain Axis in Infants and Children

Until recently, the knowledge about development of gut microbiota in children was that the complete population was set at one year of age. Modern research possibilities have proven that the development takes place gradually in early life, in sequences of successive exposures that build up quantitative and qualitative features of the microbiome [55].

The first step in early life microbiota development resides in the pre-natal period. The broad knowledge is that the fetal GI tract is sterile at birth but some studies are suggesting that some placental contamination could be possible. Furthermore, the mode of delivery is essential for the first step large colonization. Vaginal delivery assures primary contact with bacterial strains as the infant is exposed to maternal vaginal microbiota. In cesarian delivery mode, the exposure is rounded and the first bacterial contact takes place through the skin, promoting Staphylococcus strains and environmental contamination. Moreover, in the first weeks of life, C-section born infants develop decreased populations of Bifidobacterium or Lactobacillus. As such, from post-natal infections to predispositions to diabetes, obesity and autoimmune disorder, C-section seems to be the coincidental item that appears in all these patients and therefore, the connection between delivery mode, microbiome and certain pathologies through life, is taken into consideration in the scientific community [56].

Macronutrients and micronutrients are extremely important during pregnancy but also in the peri-natal period and post-partum. From the mother’s diet to her metabolic features, quality and composition of breast milk, or external intake of processed milk, to the quality and timing of diversification, there are important periods that increase or decrease the microbiome’s quality and further gut–brain features. [57]. Another preclinical proof of direct connection between microbiome and brain development in infancy refers to the blood–brain barrier. Typically, the blood–brain barrier closes its permeability during early pregnancy. In infant germ-free mice, it has been revealed that the permeability of the blood–brain barrier remains higher than normal afterbirth, but decreases the permeability after gut microbial recolonization [58]. These results are based on a large amount of clinical observations in the area of microbiome-related neurodevelopmental disorders, specifically regarding autistic spectrum disorders [59].

The first stages of gut colonization regarding more Bifidobacterium and Enterobacteriaceae species are followed by progressive anaerobe growth and a general population development between the first and third year of life, due to diversification. The proportional evolution of the microbiome is constantly adjusting during childhood in order to ensure nutrient and enzymatic metabolism, vitamin synthesis and basal growth features [60]. In terms of neurodevelopment related to the gut microbiome, an interesting study revealed that administering *Lactobacillus rhamnosus* to 6-month-old infants, with constant follow-up in the next 13 years, revealed a significant lower incidence of developing autistic spectrum disorders or ADHD [61]. Animal model studies have begun to demonstrate the link between microbiome alterations and future neurocognitive disruptions as early life dysbiosis could alter amyloid physiology and increase risk of Alzheimer disease development in future adults [62]. Regarding mood and affection, preclinical studies have revealed the importance of microbiome efficient development in early life as it is critical in signaling neuronal circuits involved in motor control, anxiety and depressive susceptibility and social behavior in a sex-dependent manner, favoring the male groups [63].

Given the specific gut microbiota characteristics and the neurobiological modifications connected to social and emotional high impact during the pubertal period, the modulation of the gut–brain axis at this age requires further research. In particular, probiotic and prebiotic supplementation during this stage tends to have positive perspectives in improving behavioral and emotional disorders, individually or combined with certain anti-depressants, as studies have shown. More investigations into dysbiosis diagnostics and treatment should be conducted in children and adolescents in order to completely understand the impact that the digestive environment has on the neurocognitive and behavioral development [64].

In the pediatric population, functional gastrointestinal disorders require complex and multidisciplinary approaches as they influence the development and life quality of children in a sensitive period. The gut–brain axis and microbiome has been in the clinical research light as spectrum disorders have been recently linked to dysbiosis and digestive infections during infancy. The interesting highlight is that functional digestive symptoms seem to appear frequently in autistic patients and the severity of digestive manifestations seem to be connected to the severity of psychopathological manifestations. Moreover, treating dysbiosis seems to attenuate behavioral and mood symptoms. Although knowledge in this direction is still emerging, behavioral, mood and cognitive impairment in children could have a strong linkage to functional gastrointestinal disorders [65,66].

### 4.1. Infant Colic

Infant colic is a distinct entity within functional digestive disorders in children. More attention has been shed on the subject as the importance of the microbiome and gut–brain axis appears to be higher than previously thought. ROME IV criteria define infant colic as frequent and long periods of infant crying, agitation or irritability that occurs without apparent cause and cannot be treated by usual therapies. The symptoms appear in infants less than 5 months old with no evidence of fever or illness [67]. Infant colic is a relatively characteristic behavior in the first 5–6 weeks of life. Still, severity of colic may be associated with increased risk of developing functional abdominal pain disorders or allergic disorders later in childhood [68,69]. In an attempt to find answers for infant colic, a study focusing on gut microbiota of colic infants revealed that in colicky groups, there is a high abundance of anaerobic Gram-negative bacteria with a propensity toward inflammatory and gas-producing activity. The Proteobacteria population was higher and the Lactobacillus species were poorly represented in comparison with non-colicky infants [70,71]. As such, probiotic therapies with Lactobacillus strains have been proposed for studies. A recent meta-analysis has revealed that Lactobacillus can significantly reduce the crying periods via possible anti-inflammatory effects or competition with anaerobic bacteria but a clear connection between infant gut microbiome and infant colic has not yet been clearly established and further clinical studies are required [72].

### 4.2. Antibiotics in Functional Gastrointestinal Disorders as Modulator of Microbiome–Gut–Brain Axis in Infancy and Childhood

Unlike adulthood, where gut microbiota can be adjusted with the incidence of fewer long-term effects, the matter is more complex in infants and children. Although there is knowledge of several FGID connected with bacterial overgrowth that can be adjusted via antibiotics such as rifaximin, special care must be taken into account when dealing with a child’s microbiome [73]. Studies show that not only antibiotic therapies during the post-natal period and in early life can promote future FGID in later childhood and adulthood, but also neurodevelopmental disorders, behavioral and mood disruptions are more susceptible to appear later in life. Even more so, Alzheimer’s Disease is also supposed to have connections to antibiotic-altered microbiome in childhood, resulting in high amyloid plaque depositing [74] There are several studies that target modulation of microbiome–gut–brain axis in children through antibiotics by analyzing cocktail administrations of broad-spectrum antibiotics and specific antibiotics in different age groups and highlighting the blood–brain penetration capacity of those antibiotics as a direct or indirect influencing factor on the gut health and mental health [75,76]. In the majority of the cases, antibiotics produced important dysbiosis, altered metabolic pathways, promoted gut inflammation, decreased the microglial developmental rate and levels of brain-derived neurotrophic factor and induced cognitive, behavioral and mood impairments, resulting in a double susceptibility to neuropsychiatric disorders and FGID, in later childhood and adulthood [77,78].

## 5. Functional Digestive Disorders—The Microbiome, the Enteric System and the Brain

Given the physiology of the bidirectional mechanism of the gut–brain axis, the pathophysiology will work in the same directions. Clinical observations and hypotheses about the microbiome–gut–brain axis have been made by analyzing pathologic features from both gastrointestinal and central nervous system appearing simultaneously in patients with functional digestive disorders. However, as stated above, the interaction between brain, GI tract and microbiome is a multilateral signaling mechanism that receives influences from the environment and responds through all its relays. Moreover, the microbiome tends to be the most important moderator of the whole mechanism, acting as a dynamic organic system and ensuring the integrity of the gut–brain balance [79].

The bidirectional influence between gut and brain responds and receives information from the external environment. Given this complex pathophysiological mechanism, we can state that disbalances along the axis are part of the bio–psycho–social dynamic. This is because of all the gut-to-brain and brain-to-gut communication pathways but also because of the interference of social, professional and emotional stress involvement in this pathology, beginning with early life periods and the prominent long-term way they affect the mechanism [80].

Although the gut–brain axis and the microbiome implications in functional gastro-intestinal disorders (FGID) are currently on the focus of preclinical and clinical studies, there is still a substantial amount of information missing, regarding specific neurologic, digestive, hormonal and biochemical activity that led to these functional alterations. First of all, gut microbiota composition and richness is the primary control entity of the intestinal environment and because of that, its involvement in functional digestive disease has to be taken into consideration [81]. Taking into account the manifestations of FGID, the microbiota’s relevance to the case becomes more highlighted. GI tract motility has mutual interaction with the microbiome as increased bacterial population and specific strains may rise GI motility but also accelerated transit may alter the luminal conditions for bacterial growth and decrease its population. Additionally, microbial metabolites and bacterial-derived enzymatic activity, such as hydrogen sulfide, bile acids and short chain fatty acids, may alter GI tract motility and moreover, diet patterns influence these reactions as some diets enhance fatty acid production or contain cholecysto-kinetic ingredients that raise bile acid production [82].

Organic sensitivity and nociceptive sensibility can play a role in pain perception in FGID and high sensitivity to mechanical and chemical local stimuli has been observed in irritable bowel syndrome or functional abdominal bloating. As animal studies show, visceral sensitivity can be transferred between mice if microbiota from irritable bowel syndrome patients is transferred to germ-free mice Additionally, Lactobacillus strain therapy resulted in reduced pain and nociceptive perception in FGID patients [83].

Permeability of the GI tract wall serves as a confluence area between microbiome environment and internal medium, serving as a barrier against infections but also as a sustainable base for the healthy bacteria growth. The permeability of the intestinal wall serves the purpose for nutrient absorption. The damage of the intestinal wall barrier has been linked to FGID directly through the microbiome as some strain overgrowth can disrupt the junctions in the intestinal wall [84]. Moreover, the mucus layer serves as feeding and ligand for bacteria and it depends on fibers in the diet. Therefore, disruptions in the mucosal layer can result in microbial population decrease because of lack of their nutrient necessary. In addition, intestinal wall damage can result in overgrowth of pathogenic bacteria, inflammation and infections [85].

Gastroenteritis and other infectious pathologies of the GI tract have been linked to an average of 10% of the FGID cases, that appear in the postinfectious period and have long-term evolution. This observation reinstates the role of gut microbiome in the activity of the enteric immune system [86].

Neuro-gastroenterology and its psychiatric collaterals, as an emerging clinical domain, may need a complex interdisciplinary approach with specific management targeting the delicate connection among the gut, brain and microbiome. For example, autistic spectrum disorders in children have a high prevalence of FGID and probiotic therapy as adjuvant alleviates both digestive and psychological symptoms [87]. Germ-free mice tend to exhibit severe anxiety-like behavior and high corticosterone levels that improve after colonization with Bifidobacterium [88].

### 5.1. Irritable Bowel Syndrome

When speaking about FGID, the most characteristic pathology remains as irritable bowel syndrome (IBS). ROME IV criteria describe the IBS diagnostic characteristics [89]. The knowledge about the pathophysiology of IBS is still limited but the connection to the microbiome and gut–brain axis is becoming clearer as IBS appears after microbiome and/or nervous system disruptions but it can also produce long-term consequences over them.

Microbiome appears to have a critical role in the development of IBS. There are missing data about specific bacterial strains that could be involved in the pathophysiology of IBS but currently, there are studies that describe general population tendencies such as higher population of Firmicutes, Streptococcus and Ruminococcus but decreased Lactobacillus and Bifidobacterium strains [90]. Animal model studies have revealed that the IBS symptoms are transferable which means the influence over the brain could be top–bottom or bottom–top in the original group but in the transfer group, appearance of neuropsychiatric symptoms is related to a bottom–top mechanism. As such, germ-free mice have been colonized with IBS patient microbiota which resulted in faster transit, higher intestinal wall permeability, increased immune cell infiltration and also, developed anxiety and depressive-like behavior, proving the complex bidirectional co-ordination between gut and brain, managed by basal microbiome functions [91]. Human studies in the form of gut–brain axis research are still incipient but the actual literature reveals some promising hypotheses based on probiotic intervention studies. As such, Bifidobacterium colonization of GI tract of IBS patients resulted in decreased inflammatory activity, lower pain perception and improved mood disruptions [92]. Another study showed the importance of an efficient management of IBS as a long evolution of the pathology could result in increased susceptibility for neurodegenerative disorders with cognition disruption starting early. The pathophysiology of this linkage is related to amyloid metabolism [93].

Depression symptoms tend to appear more prominent in FGID patients and especially with IBS patients than in non-FGID patients and alterations, with higher anxiety and depression HAMD-scores. In addition, MRI investigation in FGID patients and non FGID-patients proved that alteration in the brain structures that process emotion, behavior and cognition exist simultaneously and independently of digestive symptoms, although the mutual influence appears to sustain the severity of both syndromes [94].

In children, microbial profiling of IBS patients revealed that subtypes of the disorder are characterized by different bacterial families. In IBS with predominant constipation there tends to be increased population of Bacteroides and Haemophilus and in IBS with diarrhea predominance, there are higher populations of Lactobacillus and Bifidobacterium [95]. IBS in children has been related to many psychiatric disorders such as ADHD, autistic spectrum disorders, anxiety, therapy-resistant depression and neuro-developmental disorders. Most studies involve probiotic therapies with promising results in improving symptoms and life quality in these children and therefore, the actual focus on understanding more of the gut–brain mechanism involved in these pathologies. Until now, *Lactobacillus rhamnosus* and reuteri have had the most positive results in improving psychiatric disorders in gastrointestinal clinical features, especially with positive effects on abdominal pain, emotional and cognitive impairments [96].

### 5.2. Functional Abdominal Pain

Abdominal pain accompanies much of the FGID and it is usually used as a marker of symptom severity, although given the connections of FGID with psychiatric symptoms, some mood disorders could increase the pain perception, independently of the organic pathophysiology. Studies on functional abdominal pain, with no selectivity for specific functional digestive disorders, have been conducted in order to separate abdominal pain from other GI symptoms. In children, with functional abdominal pain, for example, gut microbiota examination showed a distinctive alteration in the Firmicutes/Bacteroides ratio in favor of Firmicutes. These data appeared in all patients, regardless of the functional digestive disorder; therefore, it opens a diagnostic perspective in that direction [97]. Still, objective connections between functional abdominal pain and psychiatric symptom scores should further be investigated for an exhaustive analysis.

### 5.3. Functional Abdominal Bloating

Functional abdominal bloating refers to recurrent bloating or distension occurring at least once a week and predominates over other symptoms but with no criteria for other functional digestive disorders and no connection to organic pathology [98]. Still, bloating/distension can appear as common complaints amongst FGID patients and most of the clinical observations have been made on patients with IBS and bloating symptoms. The involvement of gut microbiome can easily be suspected in these cases and recent studies have showed that rifaximin treatment improved bloating in patients with IBS [99]. Probiotic therapy is poorly studied but there are some observations that suggest that Bifidobacterium strains can reduce bloating in IBS patients, which confirms that competitional colonization, bacterial overgrowth and fermentative effects on the visceral sensory system are involved in abdominal functional bloating [100]. In children, there is even less information on functional bloating but again, Lactobacillus strains seem to be beneficial in reducing distension and the perception of pain in pediatric patients who present with this complaint [101].

### 5.4. Functional Constipation

Roma IV criteria for functional constipation (FC) include straining, hard stools, incomplete evacuation sensation, anorectal obstructive sensation during more than 25% of defecation, necessity of manual maneuver to facilitate stool, rare loose stools and no criteria for irritable bowel syndrome [102]. In additional, psychological comorbidities are present in significant percentages of patients with FC, confirming yet again that more than environmental factors and disability influence gastrointestinal disorders [103]. Another interesting study revealed that fecal matter transplantation in patients with primarily psychiatric symptoms and functional digestive symptoms (IBS, FC and functional diarrhea) improved both psychiatric HAM scores on anxiety and depression but also the digestive symptoms, possibly by enhancing microbiota diversity [104]. Functional constipation in children has little evidence regarding microbiome and the gut–brain axis. There are some studies that suggest Bacteroides and Lactobacillus strain colonization could increase stool frequency in constipated children, but given the special features regarding children’s gut microbiome, there are still many angles to cover through research [105]. Some observational studies suggest a linkage between functional constipation in older children and social and emotional factors, followed by behavioral, attention and memory deficits and mood disorders. Moreover, diet patterns seem to play much bigger roles in children’s functional constipation than in adults [106].

### 5.5. Functional Dyspepsia (FD)

Regarding the microbiome and gut–brain axis connection with FD, there are still few studies that could provide evidence in this direction, yet perspectives remain open. A recent study has been conducted on FD patients by 16rDNA gene sequencing of duodenal microbiome. Results showed significant strain differences between FD patients and healthy groups especially regarding species such as Staphylococcus, Faecalibacterium, Sutterella and Corynebacterium. In addition, microflora functions were altered in the FD group showing that the ureolysis and fumaric acid respiratory function of duodenal bacteria was significantly different than in the healthy group [107].

The interplay between duodenal flora, FD and nervous system is still debatable. Anxiety, depression, behavioral disorders and somatization are symptoms that appear as comorbidities to FD or as subsequent clinical features. An older study conducted in a hospital, analyzed the effectiveness of psychotherapy in patients presented for FD symptoms. The study revealed an improvement in dyspeptic symptoms and also in psychological scores at 1 month and 12 months follow-up after brief psychotherapy [108].

Probiotic therapy had some positive outcomes for dyspeptic symptoms in clinical studies, by changing the gastric fluid’s microbial composition to resemble the one in healthy persons [109]. Another study with a larger cohort of patients divided FD patients into two groups: post-prandial distress syndrome and epigastric pain syndrome. The two groups were then divided into a probiotic administration group and a probiotics combined with pharmacological therapy administration group. Probiotics alone showed much better improvements in symptoms than combined therapy and post-prandial distress syndrome patients showed more improvement than the epigastric pain syndrome patients [110]. On the other hand, rifaximin therapy was also beneficial in improving FD symptoms in a randomized clinical trial [111].

To date, there is no sustainable clinical evidence regarding microbiome and FD connections in children’s FGID. Small group studies have observed that no important differences in symptom severity resulted from probiotic administration but some beneficial effects were observed in the symptoms frequency in comparison with control group [112]. In the matter of modulating the gut–brain axis in functional dyspepsia, there are some studies that propose the use of antidepressants and/or antipsychotics in treating psychiatric patients with FD, focusing on tricyclic antidepressants and antipsychotics in specific combinations (flupentixol and melitracen). Results confirm the positive modulatory effect of psychiatric therapy on the gut–brain axis in FD patients but further information needs to be further revealed [113].

## 6. Pharmacological Modulation of the Microbiome–Gut–Brain Axis

In the light of the continuous evolution of the research on the gut–brain axis and the microbiome, there is a proportional evolution of neuro-gastroenterology. The target of the research should focus on finding the best therapeutical options that modulate the interconnection of the axis and its functions and not every relay, separately.

### 6.1. Antibiotics in Microbiome Management of FGIDs

Antibiotic intervention could be a key therapy in some FGID as long as bacterial overgrowth can be demonstrated. The effectiveness of rifaximin has been demonstrated through a study that showed it attenuated symptoms related to small intestinal bacterial overgrowth diagnosed by breath hydrogen testing in post-cholecystectomy syndrome patients. Further studies have demonstrated the use of rifaximin in some FGIDs, especially in IBS patients with diarrhea symptoms and abdominal bloating. Correlated to bacterial overgrowth, antibiotics could provide some therapeutic solutions. [114]. Still, they can also be the cause of microbiome disbalance and could, in theory, negatively affect the clinical evolution of FGID’s. Probiotic prophylactic supplementation could have protective effects in that area but further specific studies are needed in order to provide concrete data.

### 6.2. Prebiotics, Probiotic or Psycho-Biotics?

#### 6.2.1. Prebiotics

Given the state-of-the-art in gut microbiome importance within the gut–brain axis, there is a special interest for probiotic therapy in the modulation of both FGIDs and mental health. Although the majority of studies reveal positive perspectives in that direction, they must still be studied as diagnostics of specific bacterial dysbiosis, and individual linkage to gut–brain processes are still in phases of preclinical study or there is limited information. Prebiotics alone have been studied in both children and adult FGID but no significant improvements in symptomatology have been demonstrated apart from growth stimulation of Bifidobacterium strains which could be an indirect benefit [115]. Still, probiotics could worsen IBS with diarrhea, again demonstrating the possible bacterial overgrowth as the subsidiary mechanism [116]. As such, prebiotics need further study and careful adjustments when administered as the balance between benefits and negative effects is sensitive.

#### 6.2.2. Probiotics

The significant effect of probiotics in preclinical and clinical studies on neuropsychiatric patients as well as in FGID has raised the hypothesis that probiotics could act on both fronts and improve GI disorders and mental health proportionally. On the one hand, beneficial effects have been noted in probiotic treatments of psychiatric symptoms, especially anxiety, depression and cognitive impairments. Additionally, promising results are now emerging in the literature that link microbiome to neurodegenerative disorders and some psychotic symptoms [117,118]. On the other hand, microbiome’s connection to gastrointestinal disorders and especially functional ones is being demonstrated in the medical literature, as stated above. The coincidental factor that appears in both FGID and anxiety/depression is the Lactobacillus and Bifidobacterium strain therapeutical benefits and as such, these species could be the modulating entities that act on both the GI tract and brain. Some studies have even assessed the pathophysiologic mechanisms on which probiotics might function [119]. The studies suggest that probiotics promote luminal homeostasis which decrease neuronal hyperactivity in the amygdala, hippocampus and hypothalamus, promote neuronal growth and decrease the stress hormone level and by that, visceral hypersensitivity, in addition to providing GI symptom improvement [120,121]. In light of these observations, the term of “psychobiotics” emerged in the literature, as characteristic treatment in neuro-gastroenterology and being defined as microbial strains that specifically act on both GI tract and brain by modulating the gut–brain axis (Table 1). The terms refer to Lactobacillus, Bifidobacterium and newly, Saccharomyces strains, and studies are approaching complex knowledge and perspectives on these therapeutical options [122].

Studies on probiotics in children’s pathology have so far revealed contrasting results. IBS, functional abdominal pain and infant colic have been improved with Lactobacillus treatment, particularly showing a reduction in pain severity [123]. In addition, the combination of Bifidobacterium and Lactobacillus probiotic strain products such as VSL#3, had significantly improved IBS symptoms in children [124]. A study has been conducted on children with chronic constipation by the administration of Lactobacillus strains in one group and magnesium oxide in the other. The improvements were notable in both groups with slightly more beneficial effects in the probiotic groups but more rapid improvement seen in the magnesium group [125]. Another similar study confirmed the anterior results but made the observation that magnesium oxide alone could cause slight dysbiosis at some point [126]. When speaking about functional dyspepsia, probiotics alone seem to be less effective than combined with probiotics, as a recent meta-analysis has shown, but more data are needed as there are other treatments for gut–brain axis modulation in current studies that seem more effective in this pathology [127].

An interesting intervention for the modulation of the gut microbiome seems to be in fecal matter transplant. Given the possibilities of symptom transfer in animal models and human studies, the possibility of a backward beneficial therapy is not surprising. Although human studies were conducted on small groups, there seems to be positive results which open new perspectives. An interesting study analyzed fecal matter transplant effects on patients with both FGIDs and psychiatric symptoms using ROME criteria and HAMD anxiety and depression scores. A significant improvement in both GI and psychiatric symptoms was revealed at 4 weeks follow-up. The result encourages more research into this trend [128].

**Table 1:** Bacterial strains with potential “psychobiotic” usage in functional digestive disorders.

Bacterial Strain	Gastrointestinal Effects	Psychiatric Effects	Source
*Lactobacillus casei*	Anti-inflammatory effect, pain relief (children and adults)	Anxiety and depression symptom improvement, cognitive improvement	[129,130]
*Lactobacillus* *acidophilus*	Motility symptoms improvement, pain reduction, distension relief (children and adults)	Potential role in modulation of cannabinoid receptors	[131,132]
*Lactobacillus brevis*	Anti-inflammatory effect, motility symptoms improvement, intestinal barrier function improvement (children and adults)	Sleep, mood and affective symptoms imrpivement (important role in ^1^ GABA regulation)	[133,134]
*Lactobacillus rhamnosus*	Pain relief, anti-inflammatory effect (especially in children) (children and adults)	Cognitive improvement, mood and affective symptoms improvements (Dopamine, Glutamate, ^2^ 5-Ht regulation)	[135,136]
*Lactobacillus helveticus*	Decrease in visceral hypersensitivity, pain relief, anti-inflammatory effect	Mood and behavior improvement (^3^ BDNF and ^1^ GABA regulation)	[137,138]
*Lactococcus lactis*	Under study—possible anti-inflammatory effect	Depression and anxiety score improvement (^1^ GABA regulation)	[139,140]
*Saccharomyces boulardi*	Inhibitory effect on inflammatory intestinal activity, reduces oxidative stress	Cognitive improvement	[141,142]
*Bifidobacterium longum*, *lactis*	Bloating relief, motility improvement, visceral pain relief, lactate production support via glycolysis from dietary fibers (+Firmicutes strain)	Reduces depression scores, improves cognitive and behavior symptoms (^2^ 5-HT, ^3^ BDNF regulation)	[143,144,145]
*Bacillus subtilis*	Promotes bacterial diversity, improves intestinal barrier function, inflammation resolution, improves oxidative stress	Mood, cognitive and behavior improvement (^2^ 5-HT regulation)	[146,147]
*Akkermansia muciniphila*	Inflammation resolution, intestinal barrier and enteral immune system improvement, digestive symptoms improvement	Important cognitive modulation, mood and behavior improvement	[148,149]

^1^ Gamma-aminobutyric acid. ^2^ 5-hydroxytryptamine. ^3^ Brain-derived neurotrophic factor.

### 6.3. Psychopharmacological Interventions in the Gut–Brain Axis

As neuro-gastroenterology becomes more delimitated as an interdisciplinary science, the psychiatric management of the microbiome–gut–brain axis disruption seems to be a logical choice and current literature is abundant in recent studies on using psychotropic medication for treating FGID with psychiatric symptoms (antidepressants, atypical antipsychotics, Delta ligand agents). The ROME Foundation has published an exhaustive review of current possible psychiatric interventions in the treatment of FGIDs [150]. In addition, the top–bottom regulation of psychotropics offer localized or general effects as central medication can improve broad symptoms and peripheral medication can target more specifical aspects [151].

In pediatric FGIDs, serous concerns must be taken into consideration when it comes to psychotropic medication. There is limited information on the subject and also, few psychotropic drugs are approved for pediatric usage. Still, there are few studies that suggest beneficial psychopharmacological management of functional gastrointestinal disorders, especially when psychiatric symptoms appear and interfere with social, emotional and educational development of the children or altering their life quality [152].

Until now, antidepressant therapy remains an efficient choice where there is a need for psychiatric management and amitriptyline remains the antidepressant of choice. SSRI medications lack knowledge in the field of FGID in children. Overall, there is little information about the psychopharmacologic management of FGIDs in children and other therapies, such as atypical antipsychotics which are missing completely. The limited data is understandable as psychotropics need caution and extreme attention when chosen in pediatric treatments [153].

## 7. Conclusions

The interconnection between gut flora, gastrointestinal environment and the central nervous system represents a triadic complex of individual entities, each influencing the other through multiple pathways. Each environment acts as a relay that signals each alteration with the other, followed by physiological responses that will further send modified signals through the pathway. The vicious circle can only be broken by a specific and correct therapeutical intervention that will normalize those pathways and set the system back to its balance. From early childhood to adulthood, the microbiome, the gastrointestinal system and the nervous system are developing and working symbiotically; the diversity and specificity of the bacterial strains are changing from childhood to adolescence and adulthood, keeping the homeostasis of the gastrointestinal wall and protecting its neural, immunological and endocrine activity.

In the matter of FGIDs, the newest studies suggest that the majority of patients will manifest psychiatric symptoms that will negatively influence digestive symptoms. As such, there are strong scientific arguments for combined gastroenterological and neuropsychiatric management, by modulating specific bacterial strains with probiotics and prebiotics but also by applying psychiatric treatments, starting from the minimum effective dosage.

As the current literature in the field reveals, the perspectives in the gastroenterological and psychiatric management of functional digestive disorders are not only promising, but still offer many research objectives. Specific microbiome targeting and pathophysiologic modulation of the gut–brain axis still need detailed studies but the current results are already setting the new therapeutical approaches. Furthermore, the research over the microbiome and the gut–brain axis has also developed new theories involving important severe psychiatric disorders, autoimmune pathology and neurologic diseases, which means there are new paths for multidisciplinary study and medical cooperation in the near future.

## Data Availability

Not applicable.
